# New-Onset Severe Cytopenia After CAR-T Cell Therapy: Analysis of 76 Patients With Relapsed or Refractory Acute Lymphoblastic Leukemia

**DOI:** 10.3389/fonc.2021.702644

**Published:** 2021-06-30

**Authors:** Linqin Wang, Ruimin Hong, Linghui Zhou, Fang Ni, Mingming Zhang, Houli Zhao, Wenjun Wu, Yiyun Wang, Shuyi Ding, Alex H. Chang, Yongxian Hu, He Huang

**Affiliations:** ^1^ Bone Marrow Transplantation Center, The First Affiliated Hospital, School of Medicine, Zhejiang University, Hangzhou, China; ^2^ Institute of Hematology, Zhejiang University, Hangzhou, China; ^3^ Zhejiang Province Engineering Laboratory for Stem Cell and Immunity Therapy, Hangzhou, China; ^4^ Liangzhu Laboratory, Zhejiang University Medical Center, Hangzhou, China; ^5^ Clinical Translational Research Center, Shanghai Pulmonary Hospital, Tongji University School of Medicine, Shanghai, China

**Keywords:** acute lymphoblastic leukemia, chimeric antigen receptor, cytokine release syndrome, severe cytopenia, hematopoietic recovery, prolonged hematological toxicity

## Abstract

Although chimeric antigen receptor T (CAR-T) cell therapy has proven to be effective in treating relapsed or refractory B-cell hematological malignancies, severe hematological toxicities remain an intractable issue. This retrospective study assessed the characteristics and risk factors of new-onset severe cytopenia following CAR-T cell infusion in 76 patients with r/r acute lymphoblastic leukemia. The rates of new-onset severe cytopenia were high, including severe neutropenia (SN) (39/56, 70%), severe anemia (SA) (35/66, 53%), and severe thrombocytopenia (ST) (31/64, 48%). Comparatively, cohorts with higher cytokine release syndrome (CRS) grades had higher incidence of severe cytopenia with prolonged duration. Multivariable analyses showed that elevated maximum (max) lg D-dimer and delayed peak time of CRS are independent risk factors for SN recovery; increased max lg IL-10 and delayed CRS recovery are risk factors for SA; high max lg ferritin is a risk factor for ST; and longer period to CRS onset or CRS recovery and higher grade of CRS are risk factors for prolonged hematological toxicities. These observations led to the conclusion that profiles of CRS, including its duration, severity and serum markers are correlated to the incidence and recovery of new-onset severe cytopenia, prompting clinical intervention for post-CAR-T severe cytopenia.

## Introduction

Although the outcome of newly diagnosed acute lymphoblastic leukemia (ALL) has been substantially improved by intensive chemotherapy regimens and novel targeted drugs, patients progressing to the relapsed or refractory (r/r) stage exhibit poor prognosis (1), with a complete remission (CR) rate of 30–45% and a median overall survival (OS) of 5–9 months (2). Further, salvage allogeneic hematopoietic stem cell transplantation (HSCT) has shown limited efficacy with a high relapse rate in this population (3, 4). Therefore, the development of novel strategies to induce CR and extend the survival of such patients is essential.

The development of chimeric antigen receptor (CAR) T cell-based therapies has dramatically revolutionized the prognosis of r/r B-cell malignancies, including ALL, non-Hodgkin lymphoma, and multiple myeloma (5). CARs, recombinant surface proteins consisting of antigen-binding and T-cell-activating domains, can direct T cells to lyse malignant cells independently of the major histocompatibility complex (6). In r/r ALL patients, the CR rate of CD19 targeted CAR-T cell therapy is as high as 70–90%, allowing subsequent HSCT and leading to long-term survival (5, 7–12). However, various adverse effects (AEs) remain unsolved, preventing the widespread use of CAR-T cell therapy (13).

Besides cytokine release syndrome (CRS) and neurotoxicity, hematological toxicity (HT) is another common AE with an incidence of higher than 90% and associated with dismal outcome (14). For instance, Sarah et al. reported that prolonged severe HT is associated with shorter 1-year OS (15). Of note, severe HT and delayed hematopoietic recovery are associated with a higher risk of infection (16), prolonged hospitalization, high medical cost (17), and decreased quality of life. However, large-sample studies on post-CAR-T severe HTs among patients with r/r ALL are relatively few, and studies performing deep exploration of the etiology as well as the underlying mechanism are lacking (14, 18).

Here, we systematically analyzed the temporal characteristics and risk factors of new-onset severe HTs among patients with r/r ALL participating in a phase 1/2 clinical trial of CAR-T cell therapy. Moreover, risk factors impacting the recovery of severe HT were carefully dissected. Additionally, we analyzed the correlation between hospitalization-costs and temporal characteristics of severe HTs. Our work is mainly intended to provide etiological clues for CAR-T-associated cytopenia for further mechanistic studies.

## Methods

### Patient Selection

A total of 86 patients with r/r ALL who underwent CAR-T cell therapy between April 2015 to September 2020 were retrospectively reviewed. All patients were enrolled in phase 1/2 open-label single-center clinical trials of CAR-T cell therapy targeting CD19 (ChiCTR-ORN-16008948, n=55), CD22 (ChiCTR1800017402, n=5), or CD19/CD22 (ChiCTR1800015575, n=26). To eliminate confounding variables contributing to severe cytopenia and impaired hematopoietic function (e.g., primary malignancy progression), patients who did not achieve CR or withdrew from the trials at 28 days were excluded from the analysis of cytopenia characteristics. This study was conducted in full compliance with the ethical principles of the Declaration of Helsinki and was approved by the ethics committee of the First Affiliated Hospital of Zhejiang University.

### CAR-T Cell Manufacture and Clinical Protocol

**Figure 1:**
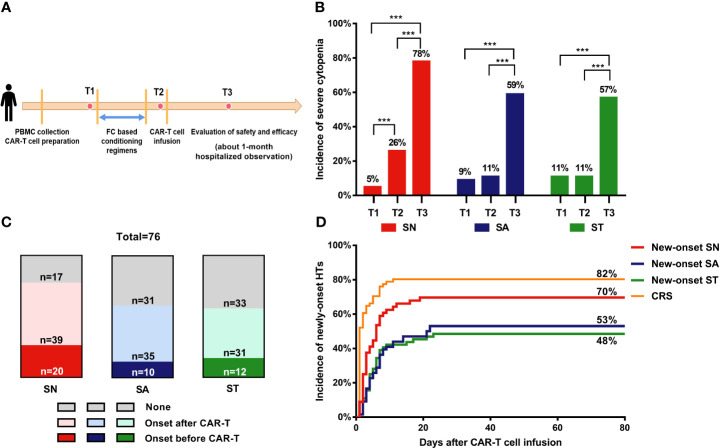
Incidence of severe cytopenia before and after CAR-T cell infusion. **(A)** Clinical protocol of CAR-T cell therapy includes lymphocyte collection, lymphodepleting chemotherapy, CAR-T cell infusion, and evaluation of safety and efficacy. **(B)** Percentage of severe cytopenia changes after lymphodepletion and CAR-T cell infusion. Two-sided P value was determined *via* Pearson Chi-square test (Bofferoni adjusted). **(C)** Proportion of severe cytopenia onset before and after CAR-T cell infusion. **(D)** Cumulative incidence of new-onset severe cytopenia and CRS. T1: before lymphodepletion; T2: interval between the end of chemotherapy and CAR-T cell infusion; T3: after CAR-T cell infusion. ***P < 0.001. CRS, cytokine release syndrome; PBMC, peripheral blood mononuclear cell; SN, severe neutropenia; SA, severe anemia; ST, severe thrombocytopenia.

Peripheral blood mononuclear cells were collected from enrolled patients for CAR-T cell generation. The protocol for CAR-T cell manufacture in our center has been described previously (19). The construct designs of CD19 and CD19/CD22 CARs in our center were reported previously (10, 20). Moreover, the single chain fragment variable of CD22 CAR was specific to recognize CD22. All their costimulatory domains were 4-1BB. Each individual was administered a single cycle of fludarabine (30 mg/m^2^ on day -4 to -2)- and cyclophosphamide (750 mg/m^2^ on day -3 to -2)- based conditioning treatment, followed by CAR-T cell infusion (**Figure 1A**). The established hospitalized observation time was 1 month, but it might change according to the severity and the recovery of toxicities. The vital signs were monitored daily. Routine blood tests were conducted and levels of serum cytokines and biochemical markers were determined, at least three times a week. Serum cytokines detected by enzyme linked immunosorbent assay included interleukin (IL)-2, IL-4, IL-6, IL-10, IFNγ, TNFα, and IL-17, while, serum biochemical markers included ferritin, D-dimer, C-reactive protein (CRP). All inflammatory markers were log transformed (marked with “lg”), and peak levels were marked with “maximum (max)”. The clinical response was evaluated 28 days after the cell infusion.

### Definitions of HT and Hematologic Recovery

The criteria for cytopenia and recovery were defined as per the Center for International Blood and Marrow Transplant Research (CIBMTR) reporting guidelines. Neutropenia and severe neutropenia (SN) were defined as absolute neutrophil counts (ANC) lower than 1.5×10^9^/L and 0.5×10^9^/L, respectively. Anemia was defined as hemoglobin concentration lower than 120 g/L in men and 110 g/L in women; levels lower than 60 g/L referred to severe anemia (SA). Thrombocytopenia and severe thrombocytopenia (ST) were defined as platelet counts < 100×10^9^/L and < 20×10^9^/L, respectively.

Neutrophil recovery was defined as ANC > 0.5 ×10^9^/L for three consecutive days, irrespective of growth factor administration. Hemoglobin recovery was defined as a hemoglobin concentration > 60 g/L without the support of erythrocyte transfusion. Platelet recovery was defined as platelet counts > 20×10^9^/L for three consecutive days in the absence of platelet transfusion. Prolonged HT (PHT) was defined as the presence of SN, SA, or ST on day 28 post infusion.

### CRS and Neurotoxicity

CRS was assessed according to a revised grading system (21). Grade 1–2 CRS was mild, while grade 3–4 CRS was severe. The assessment of neurotoxicity was based on the Common Terminology Criteria for Adverse Events 5.0 (CTCAE 5.0). CRS and neurotoxicity were assessed by three experienced clinicians; they took comprehensive factors into consideration, including clinical symptoms, vital signs, *in-vivo* expansion of CAR-T cells, levels of serum cytokine/other biomarkers, adoptions of supportive therapy, as well as infectious signs. Inconsistencies were further discussed.

### Statistical Analysis

Descriptive statistics were used to describe the patients’ baseline characteristics and the temporal profiles of severe cytopenia. Continuous variables were analyzed using the Mann-Whitney U test; the Kruskal-Wallis test was used for intergroup comparisons. Categorical variables were analyzed using the Chi-square test. Spearman correlation analysis was used to analyze the relationship between CRS/inflammatory markers and cytopenia profiles. The hazard ratio of the risk factors associated with hematopoietic recovery was determined using Cox regression analysis. A P value ≤ 0.05 (two-tailed) was considered statistically significant. Data were analyzed using the IBM SPSS Statistics version 20.

## Results

### Clinical Patient Characteristics

A total of 86 r/r ALL patients subjected to CAR-T cell therapy between April 2015 and September 2020 at the First Affiliated Hospital, Zhejiang University, were followed. Ten patients were resistant to CAR-T cells or withdrew from the study within 28 days; therefore, 76 patients were included in the analysis. The median baseline tumor burden was 31% (range, 0–96%). Fourteen (18.42%) patients had extramedullary lesions. Before the lymphodepletion regimen, patients received a median of four chemotherapy cycles (range, 1–24), and 21 (27.63%) patients received allogeneic HSCT. Fifty (65.79%) patients were treated with CD19 CAR-T cells, four (5.26%) patients were infused with CD22 CAR-T cells, and 22 (28.95%) were treated with CD19/CD22 bispecific CAR-T cells.

After CAR-T cell infusion, 81.58% (62/76) of the patients experienced CRS: 33 and 29 patients developed mild and severe CRS, respectively, with a median duration of 7 to 8 days. In the mild CRS group, 4 of 33 patients received tocilizumab or steroids. In the severe CRS group, 24 of 29 patients received tocilizumab or steroids. Moreover, 11.84% (9/76) of the patients developed mild neurotoxicity (**Table 1**).

**Table 1:** Demographic and clinical characteristics of ALL patients (n=76).

Characteristic	
**Age, years**	31.5 [15-74]
**Gender, n (%)**	
**Male**	38 (50%)
**Female**	38 (50%)
**BMI, kg/m^2^**	21.53 [14.88-30.49]
**Extramedullary disease, n (%)**	14 (18.42%)
**Number of prior chemotherapies, times**	4 [1-24]
**Prior allogeneic HSCT, n (%)**	21 (27.63%)
**Number of relapses, times**	1 [0-8]
**Bone marrow tumor burden before lymphodepletion, %**	31 [0-96]
**Pre-lymphodepletion**	
**ANC, ×10^9^/L**	2.3 [0-9.1]
**Hemoglobin, g/L**	99.5 [45-159]
**Platelet count, ×10^9^/L**	105.5 [6-412]
**LDH, U/L**	271 [135-5268]
**Ferritin, ng/mL**	836.9 [17.2-24458]
**ALT, U/L**	19 [5-449]
**AST, U/L**	21 [8-184]
**GFR, ml/min**	122 [59-172.61]
**Serum creatinine, μmol/L**	59.5 [34-97]
**Serum urea, mmol/L**	4.05 [1.4-9]
**Serum uric acid, μmol/L**	327 [89-609]
**Targets of CAR-T cell, n (%)**	
**CD19**	50 (65.79%)
**CD22**	4 (5.26%)
**CD19-CD22**	22 (28.95%)
**PHT, n (%)**	19 (25%)
**CRS, n (%)**	
**None**	14 (18.42%)
**Grade 1**	11 (14.47%)
**Grade 2**	22 (28.95%)
**Grade 3-4**	29 (38.16%)
**Neurotoxicity, n (%)**	9 (11.84%)
**Hemorrhage, n (%)**	14 (18.42%)
**Infection, n (%)**	13 (17.11%)

Data were described as n (%) or median [range].

ALT, alanine aminotransferase; ANC, absolute neutrophil count; AST, aspartate aminotransferase; BMI, body mass index; CRS, cytokine release syndrome; GFR, glomerular filtration rate; PHT, prolonged hematological toxicity.

### Incidence and Temporal Characteristics of HTs

Before lymphodepleting chemotherapy, the median ANC was 2.3 (range, 0–9.1) ×10^9^/L, the median hemoglobin concentration was 99.5 (range, 45–159) g/L, and the median platelet count was 105.5 (range, 6–412) ×10^9^/L (**Table 1**). After lymphodepletion chemotherapy, the incidence of SN significantly increased from 5% to 26% (P<0.001), while no significant changes were detected with respect to SA (9% *vs.* 11%) and ST (11% *vs.* 11%) during this short interval. After CAR-T cell infusion, the total incidence of HT increased remarkably (78% for SN, 59% for SA, and 57% for ST) (**Figure 1B**). Our dynamic observation after CAR-T cell infusion revealed that the incidence of all cytopenia reached the peak at around day 5, and reduced thereafter at different speeds (**Supplementary Figures 1C–E**); anemia persisted for a long time, whereas neutropenia and thrombocytopenia recovered soon after the peak. Regarding severe cytopenia, the incidence of SN was the highest, followed by those of ST and SA; of note, the incidence of SN and ST declined soon after, while that of SA remained stable for a month.

Considering that severe HT onset before CAR-T infusion was caused by disease progression or therapy overload, we only focused on the new-onset cytopenia to analyze the risk factors of CAR-T-related severe cytopenia (**Figure 1C**). The cumulative incidence curves showed that CRS occurred most quickly and had the highest cumulative incidence (82%), followed by new-onset SN (70%). SA and ST occurred at a similarly slow speed and had reached a relatively low incidence (53% and 48%, respectively) (**Figure 1D**).

The temporal characteristics of severe cytopenia and new-onset severe cytopenia are summarized in **Supplementary Tables 1** and **2**, respectively. No significant differences were found among three-lineage aplasia in terms of the onset time, recovery time, and duration. However, we found that the peak times of both SN and new-onset SN were the shortest, in line with the previous description (**Supplementary Figures 1A, B**).

Furthermore, we compared the temporal profiles of severe cytopenia developed before and after CAR-T cell infusion (**Supplementary Figures 2A–C**). All types of new-onset severe cytopenia reached the nadir of hematologic parameter relatively slower than those with baseline severe cytopenia. As for hematopoietic recovery, new-onset ST was more easily restored than those with baseline severe cytopenia (P=0.028). Of note, and expectedly, severe cytopenia conditions before CAR-T cell infusion had a significantly longer duration than the new-onset counterparts.

### Factors Associated With the Incidence of Severe HT

Next, we analyzed the patients’ characteristics, prior therapies, and CAR T-cell-therapy-associated factors to identify the factors correlated to the new-onset severe cytopenia.

Univariate analyses revealed that the baseline bone marrow tumor burden, CRS severity, several serum biomarkers (including max lg CRP, IL-10, IFNγ, ferritin, and D-dimer levels), and the usage of tocilizumab/corticosteroids were statistically associated with the incidence of severe cytopenia (**Supplementary Tables 3**–**5**). Moreover, durable CRS was recognized as a risk factor for both SN and SA, high max lg IL-2 for both SN and ST, high max lg IL-6 for both SA and ST, high baseline lg LDH for SN, and high max lg IL-4 for SA. Most of the above factors suggest that the incidence of severe cytopenia is mainly associated with CRS. Therefore, we explored their correlation in further detail.

#### CRS Correlates With the Incidence of Severe HT

**Figure 2:**
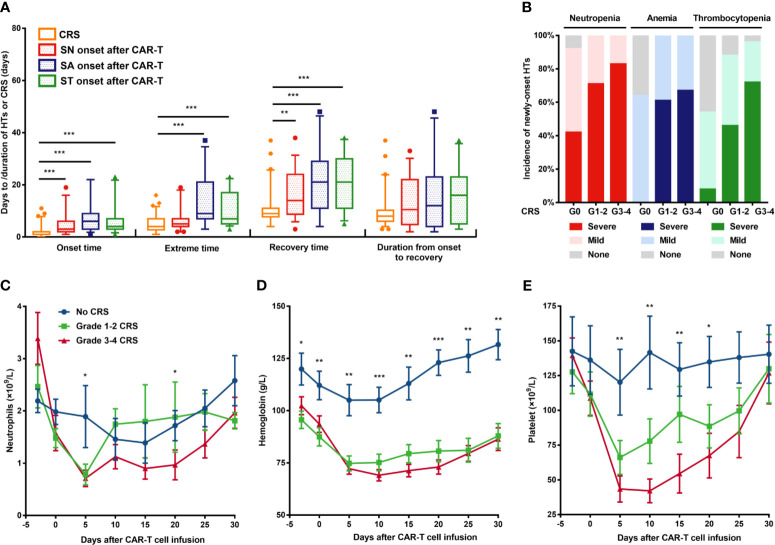
New-onset severe hematological toxicities correlated with CRS. **(A)** Comparison of temporal characteristics between CRS and severe cytopenia. **(B)** Incidence of each cytopenia is shown in patients without CRS or mild or severe CRS. **(C–E)** Neutrophil count, hemoglobin concentration, and platelet count (means ± SEMs) are shown at the indicated time after CAR-T cell infusion in patients without CRS or with mild or severe CRS. P values were determined using the Mann-Whitney U test (for **A**) or the Kruskal-Wallis test (for **C–E**). ***P < 0.001, **P < 0.01, *P < 0.05. CRS, cytokine release syndrome; HT, hematological toxicity; SN, severe neutropenia; SA, severe anemia; ST, severe thrombocytopenia.

CRS occurred, peaked, and recovered significantly ahead of severe cytopenia (**Figure 2A**). Moreover, the grade of CRS is significantly higher among patients with new-onset severe cytopenia than those without severe cytopenia (P=0.043 for SN, 0.001 for SA, 0.001 for ST) (**Supplementary Tables 3**–**5**). As the severity of CRS increased, the incidence of cytopenia and severe cytopenia showed an obvious upward trend (**Figure 2B**). Therefore, we decided to divide patients into three groups based on the CRS grade and to assess the hematological parameters separately. The ANC reached the maximum in both the mild CRS and severe CRS cohorts at day 5, but were quickly restored to approximately normal levels in the mild CRS cohort in the next 5 days, while the levels in the severe CRS cohort showed a longer restoration time (**Figure 2C**). Conversely, the fluctuation of hemoglobin concentration was similar between the mild and severe CRS cohorts (**Figure 2D**). Additionally, in the severe CRS group, the platelet count decreased more sharply than that in the mild CRS group; however, both groups recovered at a similar speed (**Figure 2E**).

#### Inflammatory Factors Correlate With the Severity of HT

**Figure 3:**
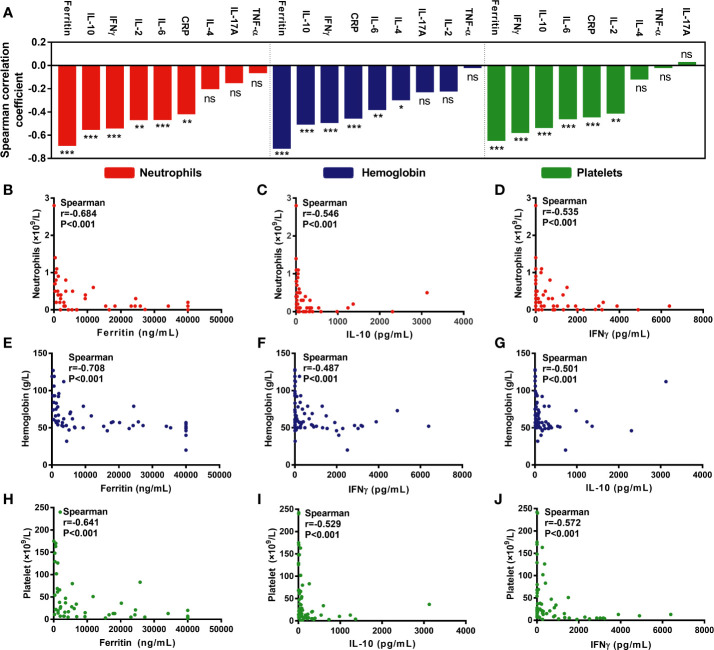
Hematological parameters are correlated with cytokines/serum biochemical markers associated with CRS. **(A)** An overview of the correlation between minimum hematological parameters after CAR-T cell infusion and peak cytokines or serum biochemical markers associated with CRS. **(B–J)** Max lg ferritin, IL-10, and IFNγ were most significantly associated with hematological parameters, including neutrophil count **(B–D)**, hemoglobin concentration **(E–G)**, and platelet count **(H–J)**. P values and r values were determined by Spearman correlation analysis. ***P < 0.001, **P < 0.01, *P < 0.05, ns, no significance.

CRS is associated with remarkable changes in the levels of cytokines and serum biochemical markers (22). Therefore, we analyzed the correlation between the nadir values of hematological parameters and the peak values of nine cytokines/serum biochemical markers (**Figure 3A**).

As per the correlation coefficient ranking, max lg ferritin, IFNγ, and IL-10 were the most important factors negatively associated with the minimum ANC (**Figures 3B–D**), hemoglobin (**Figures 3E–G**), and platelet counts (**Figures 3H–J**). Moreover, max lg IL-6 and CRP, with lower r values, were also negatively associated with the above parameters. Besides, max lg IL-2 was negatively associated with the minimum ANC and platelet counts, while that of max lg IL-4 was the only factor negatively correlated with hemoglobin concentration (**Figure 3A**).

### Factors Associated With the Recovery of Severe HTs

**Figure 4:**
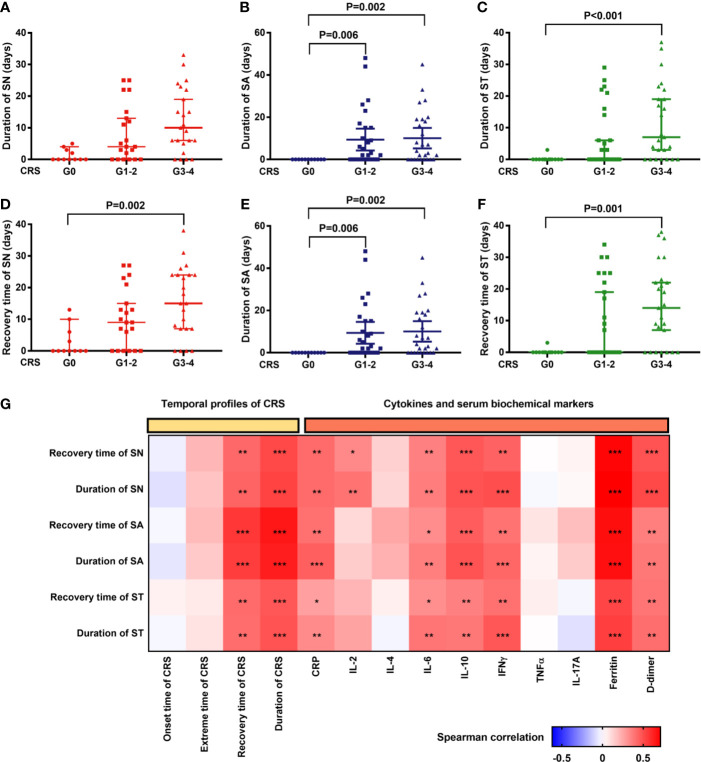
Hematopoietic recovery after CAR-T cell infusion is significantly correlated with profiles of CRS and peak cytokines/serum biochemical markers associated with CRS. **(A–C)** The duration of severe cytopenia (median and 95%CI) is shown in patients without CRS or with mild or severe CRS. The duration of patients without cytopenia after CAR-T cell infusion was defined as “0”. P values were tested using the Kruskal-Wallis test. **(D–F)** Recovery time from severe cytopenia (median and 95%CI) is shown in the aforementioned cohorts. The day of CAR-T cell infusion was recognized as the start points. P values were tested in the aforementioned way. **(G)** Correlation of profiles of severe cytopenia with profiles of CRS/serum markers. P values and r values were determined by Spearman correlation analysis. Serum markers were peak levels and log-transformed for correlation analysis. CRP, C-reactive protein; CRS, cytokine release syndrome; IFN, interferon; IL, interleukin; LDH, lactate dehydrogenase; SN, severe neutropenia; SA, severe anemia; ST, severe thrombocytopenia; TNF, tumor necrosis factor. ***P < 0.001, **P < 0.01, *P < 0.05.

Comparison of the duration and recovery time of severe cytopenia among patients in different CRS grade groups revealed that patients with CRS recovered slower than those without CRS (**Figures 4A–F**). Furthermore, in Spearman correlation, we found that delayed CRS recovery (persistent CRS) was positively correlated with delayed recovery (longer duration) of severe cytopenia (**Figure 4G**). In addition, the max lg CRP, IL-6, IL-10, IFNγ, ferritin, and D-dimer levels were positively correlated with the duration of SN, SA, and ST, while max lg IL-2 levels were only positively correlated with the duration of SN.

**Figure 5:**
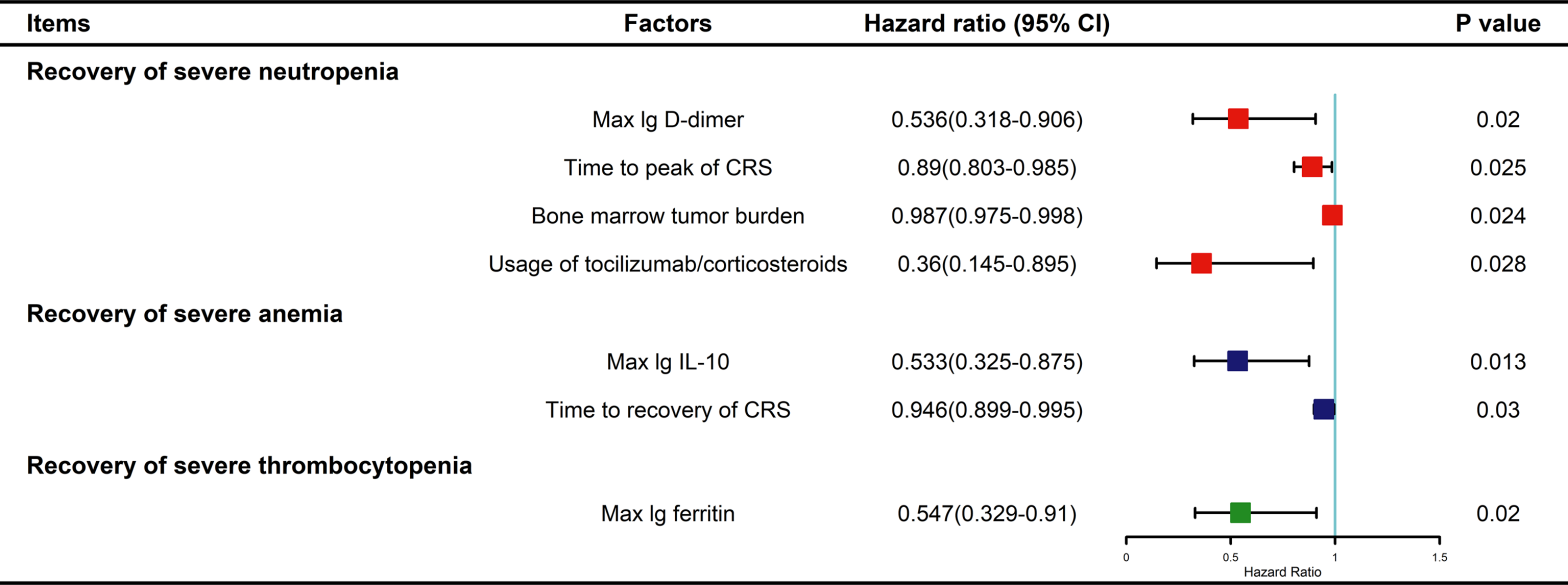
Multivariable Cox analysis for factors impacting recovery from severe cytopenia. P values were tested by Cox regression model. CRS, cytokine release syndrome; IL, interleukin.

In fact, multivariate analysis revealed that a higher baseline tumor burden (HR: 0.987; 95% CI: 0.975–0.998; P=0.024), higher max lg D-dimer level (HR: 0.536; 95% CI: 0.318–0.906; P=0.02), longer time to CRS peak (HR: 0.89; 95% CI: 0.803–0.985; P=0.025), and tocilizumab/corticosteroid use (HR: 0.36; 95% CI: 0.145–0.895; P=0.028) were independent risk factors for delayed recovery of SN (**Figure 5**). For the recovery of SA, higher max lg IL-10 (HR: 0.533; 95% CI: 0.325–0.875; P=0.013) and longer CRS recovery time (HR: 0.946; 95% CI: 0.899–0.995; P=0.03) were independent risk factors. Lastly, higher max lg ferritin level (HR: 0.547; 95% CI: 0.329–0.91; P=0.02) was an independent risk factor for delayed recovery of ST.

Furthermore, we found that longer period to CRS onset (OR: 1.317; 95% CI: 1.005-1.726; P=0.046) or CRS recovery (OR: 1.167; 95% CI: 1.028-1.325; P=0.017) and higher grade of CRS (OR: 5.312; 95% CI: 1.115-25.317; P=0.036) were independent risk factors for PHT.

### Treatment, Prognosis, and Hospitalization Cost of Patients With Severe Cytopenia

**Figure 6:**
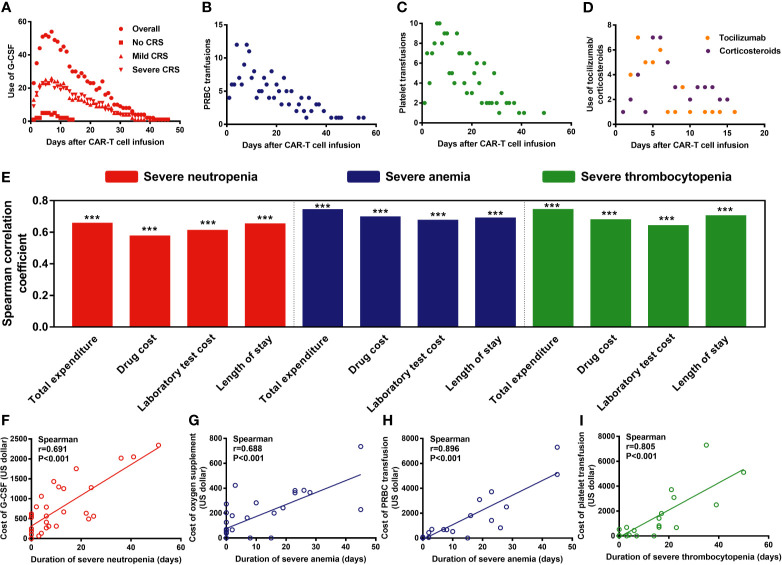
Management of severe cytopenia and the correlation between severe cytopenia and hospitalization expenses/length of stay. P values and r values were determined by Spearman correlation analysis. ***P < 0.001. G-CSF, granulocyte colony-stimulating factor; PRBC, packed red blood cells. **(A–D)** Frequencies of G-CSF administration, PRBC and platelet transfusion, and tocilizumab/ corticosteroid administration after CAR-T cell infusion. **(E–I)** The duration of severe cytopenia was strongly associated with the length of stay and hospitalization expenses.

Generally, the criteria for severe cytopenia are warning lines for the adoption of granulocyte colony-stimulating factor (G-CSF) intervention (ANC < 0.5×10^9^/L), packed red blood cell (PRBC) transfusion (hemoglobin levels < 60g/L), or platelet transfusion (platelet counts < 20×10^9^/L). Among 76 patients, 66 (86.84%) received G-CSF, 38 (50%) received PRBC transfusions, and 35 (46.05%) received platelet transfusions. The frequency of G-CSF administration peaked around days 5–7, that of PRBC transfusion peaked around days 4–9, and platelet transfusion peaked at around days 6–7 (**Figures 6A–C**). Moreover, the use of tocilizumab/corticosteroids peaked on days 5–6 (**Figure 6D**). Of note, compared with mild/severe CRS patients, patients without CRS showed a lower need for blood transfusion or G-CSF support (**Figure 6A**, **Supplementary Figure 3**).

Furthermore, we analyzed the association of PHT with other observational outcomes after CAR-T cell infusion (**Supplementary Table 8**). Hemorrhage rate was significantly higher among those patients with PHT (P=0.013), while the no significance was found in infection rate (P=0.31) and non-relapse mortality (P=0.79).

Additionally, we found that the duration of severe cytopenia was strongly associated with the length of stay and hospitalization expenses, including total expenses, drug costs, and laboratory test costs (P<0.001) (**Figure 6E**). For instance, the duration of SN was strongly associated with the cost of G-CSF (P<0.001, r=0.691) (**Figure 6F**), that of SA was strongly associated with the cost of oxygen supplementation (P<0.001, r=0.688) and PRBC transfusions (P<0.001, r=0.896) (**Figure 6G**), and the duration of ST (P<0.001, r=0.805) was associated with the cost of PRBC and platelet transfusions, respectively (**Figures 6H–I**).

## Discussion

During the management of patients with r/r ALL receiving CAR-T cell therapy, HT, in addition to CRS and neurotoxicity, is a major issue for clinicians, and persistent severe cytopenia is strongly associated with a dismal 1-year OS (15). However, only a small proportion of the literature has focused on post-CAR-T severe cytopenia. Here, to address this gap in knowledge, we aimed to dissect the characteristics and risk factors of new-onset severe cytopenia following CAR-T cell infusion, in a large cohort of patients with r/r ALL.

In this retrospective analysis, r/r ALL patients showed a high incidence of severe cytopenia after CAR-T cell infusion, consistent with the results reported in previous studies (9, 14, 23). To illustrate the effect of CAR-T therapy on hematopoietic function, we mainly focused on the new-onset severe cytopenia to minimize other confounding factors including disease progression or other treatments. The incidences of new-onset severe HTs were still high, including 70% SN, 53% SA, and 48% ST. Severe cytopenia (both overall and new-onset) of three lineages presented similar features of onset time, recovery time, and duration. Of note, SN peaked earlier than SA and ST, highlighting the need for anti-infection prophylaxis early after CAR-T cell infusion. Additionally, our results also suggest that ST is associated with a difficult recovery, which needs more attention.

Univariate analysis highlighted the bone marrow tumor burden, CRS, and CRS-associated serum biomarkers as common risk factors for SN, SA, and ST, which is consistent with the findings previously reported (14). Marrow disease burden was previously identified as an independent risk factor for a higher grade of CRS, and higher grade CRS was accompanied by higher levels of serum biomarkers (24). Therefore, probably, CRS plays an important role in the onset and progression of severe cytopenia. The supportive evidence is as follows. First, CRS occurred significantly earlier than severe cytopenia. Moreover, the incidence of severe cytopenia increased remarkably as the CRS grade increased; of interest, compared to the cohort without cytopenia, patients with new-onset SN or SA experienced more durable CRS, suggesting a temporal additive effect of CRS on hematopoietic function. Furthermore, the rate of using tocilizumab or glucocorticoids was significantly higher in cohorts with severe cytopenia. This confirms the relationship between the severity of CRS and the incidence of severe cytopenia in another way because tocilizumab and glucocorticoids are most frequently administered for patients with severe CRS in our center. Further, the minimum of ANC, hemoglobin, and platelet counts after CAR-T cell infusion were all negatively associated with the significantly elevated levels of serum biomarkers, including max lg ferritin, IL-10, and IFNγ. Consistently, all these biomarkers were previously reported to be significantly elevated in severe CRS (22, 24, 25).

As previously reported, CRS is recognized as a strong factor impacting hematopoietic recovery (15, 26); we found that CRS suppressed the recovery of the three lineages in different ways. Interestingly, though several serum cytokines were found to be correlated with the recovery of severe cytopenia *via* correlation analysis, we found that severe cytopenia of each lineage had a specific cytokine as its independent risk factor. Elevated max lg D-dimer is a risk factor for SN recovery, increased max lg IL-10 is a risk factor for SA recovery, and high max lg ferritin is a risk factor for ST recovery. These results suggest that the hematopoietic functions of the three lineages might be sensitive to different cytokines. Moreover, in line with these data, the temporal characteristics of CRS were also associated with the recovery of severe cytopenia; delayed CRS peak was found as a risk factor for SN recovery, while a delayed CRS recovery was found as a risk factor for SA recovery, suggesting that delayed CRS impacts the hematopoietic function.

The specific mechanisms underlying CAR-T-therapy-associated severe cytopenia have yet to be disclosed; currently, lymphodepleting regimens and cytokine-mediated mechanisms are the most discussed hypotheses (18). Our study revealed that lymphodepleting regimens mainly increased the incidence of SN, while the interval to CAR-T cell infusion is too short to assess its delayed toxicities. We also found that the profiles of CRS, including temporal features, severity, and levels of CRS-associated serum markers, were associated with the process of severe cytopenia. Additionally, a longer duration of cytopenia was strongly associated with elevated total expenditure and longer length of stay. Therefore, the proper management of severe cytopenia will directly benefit not only patients but also the health systems.

Our study is limited by being a retrospective single-center study, with the small sample size restricting our analysis of different CAR structures, including targets and co-stimulatory domains. Secondly, in clinical practice, the administration of G-CSF, blood transfusion, or corticosteroids would inevitably overshadow the process of HTs. Hence, to reduce the confounding factors, hematopoietic recovery, a major endpoint, was defined as the conditions without the need for growth factor administration or blood transfusion. Besides, the management strategies of severe cytopenia are unified in our center according to the guidelines. Thirdly, quantitative detection of cytokines and other serum biochemical markers need to be expanded for building an interaction network for mediating hematopoietic function or establishing predictive models of severe cytopenia.

Despite these limitations, our study, based on a relatively large sample size, provides clinical evidence for the hypothesis that CRS and serum inflammatory markers might induce severe cytopenia and impact the recovery of hematopoiesis, with implications for both clinical and basic research. Our findings could help clinicians to distinguish and pay more attention to patients with higher risk of CAR-T associated severe cytopenia. Moreover, models based on serum biochemical markers may be used to predict severe cytopenia and guide clinical interventions. Most importantly, this study supports further exploration of the mechanism of severe cytopenia after CAR-T cell infusion based on CRS-mediated regulation of hematopoiesis.

## Data Availability Statement

The original contributions presented in the study are included in the article/**Supplementary Material**. Further inquiries can be directed to the corresponding authors.

## Ethics Statement

The studies involving human participants were reviewed and approved by Ethics committee of the First Affiliated Hospital of Zhejiang University. Written informed consent to participate in this study was provided by the participants’ legal guardian/next of kin.

## Author Contributions

LW and RH collected clinical data, analyzed data, and wrote the paper. LZ, FN, HZ, and YW collected clinical data and coauthored the paper. MZ, WW, and SD analyzed the data, provided clinical care to patients and coauthored the paper. AC designed and manufactured the CAR-T cells, and wrote the paper. HH and YH designed the study, analyzed the data, provided clinical care to patients, and wrote the paper. All authors discussed and interpreted the results. All authors contributed to the article and approved the submitted version.

## Funding

This work was supported by the Natural Science Foundation of China (grant No. 81730008, 81770201), Key Project of Science and Technology Department of Zhejiang Province (grant No. 2019C03016, 2018C03016-2), Department of Education of Zhejiang Province (grant No. Y202043556), Medical Science and Technology Project of Zhejiang Provincial Health Commission (grant No. 2021432523).

## Conflict of Interest

The authors declare that the research was conducted in the absence of any commercial or financial relationships that could be construed as a potential conflict of interest.
