# Supplementary material for: New-Onset Severe Cytopenia After CAR-T Cell Therapy: Analysis of 76 Patients With Relapsed or Refractory Acute Lymphoblastic Leukemia
Source: Front Oncol. 2021 Jun 30;11:702644. doi: 10.3389/fonc.2021.702644 (PMC8278328; doi:10.3389/fonc.2021.702644)
Supplement: Supplementary file 1 [file DataSheet_1.docx]

**Supplementary materials**

**Supplementary Table Legends:**

**Supplementary Table 1.** Temporal characteristics of severe cytopenia post CAR-T cell infusion.

**Supplementary Table 2.** Temporal characteristics of new-onset severe cytopenia post CAR-T cell infusion. CRS, cytokine release syndrome.

**Supplementary Table 3.** Univariable analysis of severe neutropenia onset after CAR-T cell infusion (n=56). Data were described as n (%) or median [range]. P values were tested by Chi-Square test or Mann-Whitney U test. BMI, body mass index; CRP, C-reactive protein; CRS, cytokine release syndrome; HSCT, hematopoietic stem cell transplantation; IFN, interferon; IL, interleukin; LDH, lactate dehydrogenase; SN, severe neutropenia; TNF, tumor necrosis factor.

**Supplementary Table 4.** Univariable analysis of severe anemia onset after CAR-T cell infusion (n=66). Data were described as n (%) or median [range]. P value were tested by Chi-Square test or Mann-Whitney U test. BMI, body mass index; CRP, C-reactive protein; CRS, cytokine release syndrome; HSCT, hematopoietic stem cell transplantation; IFN, interferon; IL, interleukin; LDH, lactate dehydrogenase; SA, severe anemia; TNF, tumor necrosis factor.

**Supplementary Table 5.** Univariable analysis of severe thrombocytopenia onset after CAR-T cell infusion (n=64). Data were described as n (%) or median [range]. P value were tested by Chi-Square test or Mann-Whitney U test. BMI, body mass index; CRP, C-reactive protein; CRS, cytokine release syndrome; HSCT, hematopoietic stem cell transplantation; IFN, interferon; IL, interleukin; LDH, lactate dehydrogenase; ST, severe thrombocytopenia; TNF, tumor necrosis factor.

**Supplementary Table 6.** Univariable Cox analysis for factors impacting recovery from severe cytopenia. BMI, body mass index; CRP, C-reactive protein; CRS, cytokine release syndrome; HSCT, hematopoietic stem cell transplantation; IFN, interferon; IL, interleukin; LDH, lactate dehydrogenase; ST, severe thrombocytopenia; TNF, tumor necrosis factor.

**Supplementary Table 7.** Univariate analysis of prolonged hematological toxicities after CAR-T cell infusion. BMI, body mass index; CRP, C-reactive protein; CRS, cytokine release syndrome; HSCT, hematopoietic stem cell transplantation; IFN, interferon; IL, interleukin; LDH, lactate dehydrogenase; ST, severe thrombocytopenia; TNF, tumor necrosis factor.

**Supplementary Table 8.** Prolonged hematological toxicities with other adverse events after CAR-T cell infusion (n=76). Data were described as n (%). P value were tested by Chi-Square test. PHT, prolonged hematological toxicity.

**Supplementary Figure Legends:**

**Supplementary Figure 1.** Temporal characteristics of severe cytopenia post CAR-T cell infusion and dynamic change of incidence of cytopenia peri-CAR-T cell therapy. **(A-B)** The onset, extreme, recovery time as well as duration of severe cytopenia and new-onset severe cytopenia post CAR-T cell infusion are shown. **(C-E)** The incidences of neutropenia, anemia and thrombocytopenia are observed within a month at an interval of 5 days. Two-sided P values were determined using the Kruskal-Wallis test. HT, hematological toxicity; SN, severe neutropenia; SA, severe anemia; ST, severe thrombocytopenia.

**Supplementary Figure 2.** Comparison of temporal characteristics between severe cytopenia onset before CAR-T cell infusion and new-onset severe cytopenia. The characteristics of SN, SA, and ST were concluded in **A**, **B** and **C**, respectively. Two-sided P values were determined using the Kruskal-Wallis test. SN, severe neutropenia; SA, severe anemia; ST, severe thrombocytopenia.

**Supplementary Figure 3.** Frequencies of blood transfusions and tocilizumab/corticosteroids use among different CRS grade groups. **(A-C)** Frequencies of PRBC transfusions in different CRS groups. **(D-F)** Frequencies of platelet transfusions in different CRS groups. **(G-J)** Frequencies of tocilizumab/corticosteroids administration in mild and severe CRS groups. CRS, cytokine release syndrome; PRBC, packed red blood cells.

**Supplementary Tables**

**Supplementary Table 1. Temporal characteristics of severe cytopenia post CAR-T cell infusion**

|  | | Severe neutropenia | Severe  anemia | Severe  thrombocytopenia |
| --- | --- | --- | --- | --- |
| Days to severe hematological toxicities | | | | |
| Onset | Median | 2 | 4 | 3 |
|  | IQR | 0~5 | 1.5~7.5 | 0~6 |
|  | Range | -12~19 | -11~22 | -13~23 |
| Extreme | Median | 5 | 8 | 7 |
|  | IQR | 3~7 | 6~19.5 | 5~17 |
|  | Range | -6~22 | -11~37 | -2~23 |
| Recovery | Median | 14.5 | 20.5 | 22 |
|  | IQR | 9~26.25 | 10.25~27.75 | 11~32 |
|  | Range | 3~45 | 2~48 | 5~62 |
| Duration from onset to recovery | | | | |
|  | Median | 13 | 15.5 | 19 |
|  | IQR | 6~25 | 5.25~26.75 | 6~30 |
|  | Range | 2~51 | 2~48 | 3~67 |

**Supplementary Table 2. Temporal characteristics of new-onset severe cytopenia post CAR-T cell infusion**

|  | | Severe neutropenia | Severe  anemia | Severe  thrombocytopenia | CRS |
| --- | --- | --- | --- | --- | --- |
| Days to severe cytopenia or CRS | | | | | |
| Onset | Median | 3 | 6 | 4 | 1 |
|  | IQR | 2~6 | 3~9 | 3~7 | 1~2 |
|  | Range | 1~19 | 1~22 | 1~23 | 0~11 |
| Extreme | Median | 5 | 9 | 7 | 4 |
|  | IQR | 4~7 | 7~21 | 5~17 | 2.75~7 |
|  | Range | 2~19 | 3~37 | 3~23 | 1~16 |
| Recovery | Median | 14 | 21 | 21 | 9 |
|  | IQR | 8.75~24 | 11~29 | 11~30 | 7.75~11 |
|  | Range | 3~38 | 4~48 | 5~38 | 4~37 |
| Duration from onset to recovery | | | | | |
|  | Median | 10.5 | 12 | 16 | 8 |
|  | IQR | 4.75~22 | 4~23 | 5~23 | 6~10.25 |
|  | Range | 2~33 | 2~48 | 3~37 | 3~37 |

Abbreviation: CRS, cytokine release syndrome.

**Supplementary Table 3. Univariable analysis of severe neutropenia onset after CAR-T cell infusion (n=56)**

| Characteristic | New-onset SN (n=39) | Without SN (n=17) | P value |
| --- | --- | --- | --- |
| Age, years | 32 [15-68] | 25 [15-61] | 0.117 |
| BMI, kg/m^2^ | 21.63 [14.88-30.49] | 20.06 [15.73-26.12] | 0.433 |
| No. of prior chemotherapies, times | 4 [1-24] | 4 [1-22] | 0.653 |
| Prior allogeneic HSCT, n (%) | 10 (25.64%) | 6 (35.29%) | 0.527 |
| No. of relapses, times | 1 [0-6] | 1 [0-8] | 0.893 |
| Bone marrow tumor burden, % | 36 [0-92] | 7 [0-84] | **0.042** |
| Does of CAR-T cell, 10^6^/kg | 3.5 [0.33-12.59] | 3.2 [1.1-9.28] | 0.314 |
| Targets of CAR-T cell, n (%) |  |  | 0.488 |
| CD19 | 29 (74.36%) | 10 (58.82%) |  |
| CD22 | 1 (2.56%) | 1 (5.88%) |  |
| CD19-CD22 | 9 (23.08%) | 6 (35.29%) |  |
| Neurotoxicity | 5 (12.82%) | 1 (5.88%) | 0.44 |
| CRS grade, n (%) |  |  | **0.043** |
| 0 | 5 (12.82%) | 7 (41.18%) |  |
| 1-2 | 15 (38.46%) | 6 (35.29%) |  |
| 3-4 | 19 (48.72%) | 4 (23.53%) |  |
| Temporal profiles of CRS |  |  |  |
| Onset time, days | 1 [0~11] | 1 [0~8] | 0.516 |
| Extreme time, days | 4 [1~16] | 5 [1~9] | 0.967 |
| Recovery time, days | 9 [4~37] | 7.5 [4~12] | 0.096 |
| Duration from onset to recovery, days | 8 [3~37] | 4.5 [3~9] | **0.001** |
| Cytokines and serum biochemical markers |  |  |  |
| Max lg CRP, mg/L | 2.11 [-0.1~2.5] | 1.66 [0.18~2.26] | **0.007** |
| Max lg IL-2, pg/mL | 1.18 [-1~2.89] | 0.605 [-1~1.78] | **0.018** |
| Max lg IL-4, pg/mL | 0.55 [-1~2.43] | 0.325 [-1~1.06] | 0.444 |
| Max lg IL-6, pg/mL | 3.275 [0.98~4.66] | 2.415 [0.25~4.09] | 0.053 |
| Max lg IL-10, pg/mL | 2.175 [0.54~3.36] | 1.41 [0.57~3.5] | **0.016** |
| Max lg IFNγ, pg/mL | 2.7 [0.38~3.81] | 0.995 [-1~3.18] | **0.002** |
| Max lg TNFα, pg/mL | 0.505 [-1~4.1] | 0.475 [-1~2.65] | 0.932 |
| Max lg IL-17A, pg/mL | 0.94 [-1~1.96] | 0.82 [-1~1.89] | 0.847 |
| Max lg ferritin, ng/mL | 3.97 [2.84~4.6] | 2.91 [1.57~4.07] | **<0.001** |
| Max lg D-dimer, μg/L | 4.82 [2.29~4.95] | 3.55 [2.43~4.94] | **0.007** |
| Baseline lg ferritin, ng/mL | 3 [2.13~4.39] | 2.77 [1.24~3.47] | 0.104 |
| Baseline lg LDH, U/L | 2.44 [2.15~3.72] | 2.275 [2.17~3.43] | **0.004** |
| Usage of tocilizumab or corticosteroids, n (%) | 19 (48.72%) | 3 (17.65%) | **0.029** |

Data were described as n (%) or median [range]. P values were tested by Chi-Square test or Mann-Whitney U test. BMI, body mass index; CRP, C-reactive protein; CRS, cytokine release syndrome; HSCT, hematopoietic stem cell transplantation; IFN, interferon; IL, interleukin; LDH, lactate dehydrogenase; SN, severe neutropenia; TNF, tumor necrosis factor.

**Supplementary Table 4. Univariable analysis of severe anemia onset after CAR-T cell infusion (n=66)**

| Characteristic | New-onset SA (n=35) | Without SA (n=31) | P value |
| --- | --- | --- | --- |
| Age, years | 33 [15-66] | 27 [15-68] | 0.396 |
| BMI, kg/m^2^ | 21.50 [14.88-28.04] | 20.81 [15.73-30.49] | 0.867 |
| No. of prior chemotherapies, times | 6 [1-24] | 3 [1-22] | 0.076 |
| Prior allogeneic HSCT, n (%) | 7 (20%) | 12 (38.71%) | 0.094 |
| No. of relapses, times | 1 [0-8] | 1 [0-3] | 0.301 |
| Bone marrow tumor burden, % | 50 [0.34-96] | 7 [0-84] | **<0.001** |
| Does of CAR-T cell, 10^6^/kg | 3 [1.04-12.59] | 3.5 [0.33-9.32] | 0.946 |
| Targets of CAR-T cell, n (%) |  |  | 0.864 |
| CD19 | 24 (68.57%) | 21 (67.74%) |  |
| CD22 | 2 (5.71%) | 1 (3.23%) |  |
| CD19-CD22 | 9 (25.71%) | 9 (29.03%) |  |
| Neurotoxicity | 5 (14.29%) | 2 (6.45%) | 0.302 |
| CRS grade, n (%) |  |  | **0.001** |
| 0 | 0 (0) | 11 (35.48%) |  |
| 1-2 | 17 (48.57%) | 11 (35.48%) |  |
| 3-4 | 18 (51.43%) | 9 (29.03%) |  |
| Temporal profiles of CRS |  |  |  |
| Onset time, days | 1 [0~9] | 1 [0~11] | 0.317 |
| Extreme time, days | 6 [2~16] | 3.5 [1~13] | 0.215 |
| Recovery time, days | 9 [6~37] | 7.5 [4~17] | 0.056 |
| Duration from onset to recovery, days | 9 [3~37] | 6 [3~9] | **<0.001** |
| Cytokines and serum biochemical markers |  |  |  |
| Max lg CRP, mg/L | 2.12 [0.56~2.5] | 1.83 [-0.1~2.4] | **0.005** |
| Max lg IL-2, pg/mL | 0.845 [-1~2.89] | 0.6 [-1~1.98] | 0.147 |
| Max lg IL-4, pg/mL | 0.55 [-1~2.43] | 0.3 [-1~1.06] | **0.02** |
| Max lg IL-6, pg/mL | 3.28 [1.28~4.66] | 2.33 [0.25~4.21] | **0.004** |
| Max lg IL-10, pg/mL | 2.23 [0.75~3.36] | 1.49 [0.54~3.5] | **0.002** |
| Max lg IFNγ, pg/mL | 2.68 [0.62~3.81] | 1.84 [-1~3.69] | **0.001** |
| Max lg TNFα, pg/mL | 0.6 [-0.59~4.1] | 0.57 [-1~2.65] | 0.49 |
| Max lg IL-17A, pg/mL | 1.02 [-1~1.96] | 0.705 [-1~1.96] | 0.205 |
| Max lg ferritin, ng/mL | 4.26 [3.06~4.6] | 3.12 [1.57~4.39] | **<0.001** |
| Max lg D-dimer, μg/L | 4.9 [2.68~4.95] | 3.78 [2.29~4.94] | **0.005** |
| Baseline lg ferritin, ng/mL | 2.99 [2.13~4.39] | 2.71 [1.24~3.47] | 0.107 |
| Baseline lg LDH, U/L | 2.51 [2.13~3.72] | 2.33 [2.17~3.43] | 0.159 |
| Usage of tocilizumab or corticosteroids, n (%) | 19 (54.29%) | 7 (22.58%) | **0.009** |

Data were described as n (%) or median [range]. P value were tested by Chi-Square test or Mann-Whitney U test. BMI, body mass index; CRP, C-reactive protein; CRS, cytokine release syndrome; HSCT, hematopoietic stem cell transplantation; IFN, interferon; IL, interleukin; LDH, lactate dehydrogenase; SA, severe anemia; TNF, tumor necrosis factor.

**Supplementary Table 5.** **Univariable analysis of severe thrombocytopenia onset after CAR-T cell infusion (n=64)**

| Characteristic | New-onset ST (n=31) | Without ST (n=33) | P value |
| --- | --- | --- | --- |
| Age, years | 32 [15-66] | 30 [15-68] | 0.712 |
| BMI, kg/m^2^ | 20.00 [14.88-26.26] | 21.55 [15.73-30.49] | 0.082 |
| No. of prior chemotherapies, times | 5 [1-24] | 3 [1-22] | 0.287 |
| Prior allogeneic HSCT, n (%) | 11 (35.48%) | 10 (30.3%) | 0.659 |
| No. of relapses, times | 1 [0-6] | 1 [0-8] | 0.106 |
| Bone marrow tumor burden, % | 45 [0.34-92] | 7 [0-84] | **0.002** |
| Does of CAR-T cell, 10^6^/kg | 3 [0.33-12] | 3.5 [0.93-12.59] | 0.371 |
| Targets of CAR-T cell, n (%) |  |  | 0.447 |
| CD19 | 24 (77.42%) | 21 (63.64%) |  |
| CD22 | 1 (3.23%) | 1 (3.03%) |  |
| CD19-CD22 | 6 (19.35%) | 11 (33.33%) |  |
| Neurotoxicity | 5 (16.13%) | 2 (6.06%) | 0.197 |
| CRS grade, n (%) |  |  | **0.001** |
| 0 | 1 (3.23%) | 12 (36.36%) |  |
| 1-2 | 12 (38.71%) | 14 (42.42%) |  |
| 3-4 | 18 (58.06%) | 7 (21.21%) |  |
| Temporal profiles of CRS |  |  |  |
| Onset time, days | 1 [0~11] | 1 [0~8] | 0.699 |
| Extreme time, days | 4 [1~13] | 4 [1~16] | 0.9 |
| Recovery time, days | 9 [4~37] | 9 [4~18] | 0.475 |
| Duration from onset to recovery, days | 8 [3~37] | 7 [3~18] | 0.09 |
| Cytokines and serum biochemical markers |  |  |  |
| Max lg CRP, mg/L | 2.07 [0.56~2.43] | 1.83 [-0.1~2.4] | **0.018** |
| Max lg IL-2, pg/mL | 1.13 [-1~2.89] | 0.78 [-1~2.07] | **0.024** |
| Max lg IL-4, pg/mL | 0.55 [-1~2.43] | 0.48 [-1~1.23] | 0.797 |
| Max lg IL-6, pg/mL | 3.42 [1.28~4.66] | 2.415 [0.25~4.11] | **0.004** |
| Max lg IL-10, pg/mL | 2.235 [0.75~3.14] | 1.495 [0.54~3.5] | **0.004** |
| Max lg IFNγ, pg/mL | 2.915 [0.62~3.81] | 1.65 [-1~3.18] | **<0.001** |
| Max lg TNFα, pg/mL | 0.55 [-1~4.1] | 0.555 [-1~2.65] | 0.667 |
| Max lg IL-17A, pg/mL | 0.83 [-1~1.96] | 0.95 [-1~1.96] | 0.424 |
| Max lg ferritin, ng/mL | 4.205 [2.37~4.6] | 3.29 [1.57~4.6] | **<0.001** |
| Max lg D-dimer, μg/L | 4.9 [2.68~4.95] | 3.67 [2.29~4.94] | **0.004** |
| Baseline lg ferritin, ng/mL | 2.99 [2.13~4.39] | 2.785 [1.24~3.67] | 0.127 |
| Baseline lg LDH, U/L | 2.47 [2.15~3.72] | 2.355 [2.13~3.43] | 0.105 |
| Usage of tocilizumab or corticosteroids, n (%) | 18 (58.06%) | 5 (15.15%) | **<0.001** |

Data were described as n (%) or median [range]. P value were tested by Chi-Square test or Mann-Whitney U test. BMI, body mass index; CRP, C-reactive protein; CRS, cytokine release syndrome; HSCT, hematopoietic stem cell transplantation; IFN, interferon; IL, interleukin; LDH, lactate dehydrogenase; ST, severe thrombocytopenia; TNF, tumor necrosis factor.

**Supplementary Table 6.** **Univariable Cox analysis for factors impacting recovery from severe cytopenia**

| Characteristic | Groups | Severe neutropenia | | Severe anemia | | Severe thrombocytopenia | |
| --- | --- | --- | --- | --- | --- | --- | --- |
|  |  | **Hazard Ratio** | **P value** | **Hazard Ratio** | **P value** | **Hazard Ratio** | **P value** |
| Age, years | / | 0.993 (0.976-1.011) | 0.45 | 0.998 (0.982-1.014) | 0.787 | 1.001 (0.985-1.016) | 0.926 |
| BMI, kg/m^2^ | / | 0.998 (0.923-1.079) | 0.952 | 1.015 (0.949-1.086) | 0.664 | 1.031 (0.959-1.107) | 0.407 |
| No. of prior chemotherapies, times | / | 0.975 (0.925-1.028) | 0.356 | 0.974 (0.921-1.03) | 0.362 | 0.964 (0.911-1.02) | 0.202 |
| Prior allogeneic HSCT | Yes vs. No | 1.356 (0.748-2.458) | 0.315 | 1.324 (0.77-2.277) | 0.31 | 0.802 (0.468-1.376) | 0.423 |
| No. of relapses, times | / | 0.984 (0.812-1.194) | 0.874 | 0.879 (0.697-1.109) | 0.277 | 0.881 (0.706-1.1) | 0.265 |
| Bone marrow tumor burden, % | / | **0.987 (0.978-0.997)** | **0.01** | **0.988 (0.98-0.997)** | **0.007** | **0.99 (0.981-0.999)** | **0.028** |
| Does of CAR-T cell, 10^6^/kg | / | 0.942 (0.852-1.041) | 0.243 | 1.032(0.953-1.118) | 0.433 | 1.04 (0.954-1.134) | 0.373 |
| Targets of CAR-T cell |  |  |  |  |  |  |  |
|  | CD19 | 1 | 0.978 | 1 | 0.706 |  | 0.534 |
|  | CD22 | 0.919 (0.22-3.837) | 0.908 | 0.634 (0.195-2.062) | 0.449 | 1.467 (0.351-6.127) | 0.599 |
|  | CD19-CD22 | 0.943 (0.515-1.728) | 0.85 | 1.071 (0.619-1.855) | 0.806 | 1.361 (0.765­-2.421) | 0.295 |
| Neurotoxicity | Yes vs. No | 0.614 (0.261-1.445) | 0.264 | 0.867 (0.392-1.917) | 0.725 | 0.846 (0.383-1.867) | 0.679 |
| CRS grade |  |  |  |  |  |  |  |
|  | 0 | 1 | 0.231 | **1** | **0.026** | **1** | **0.019** |
|  | 1-2 | 0.791 (0.373-1.68) | 0.542 | **0.359 (0.164-0.785)** | **0.01** | **0.459 (0.22-0.957­)** | **0.038** |
|  | 3-4 | 0.548 (0.26-1.151) | 0.112 | **0.369 (0.168-0.807)** | **0.013** | **0.33 (0.152-0.716)** | **0.005** |
| Infection | Yes vs. No | 0.715 (0.336-1.523) | 0.385 | 0.907 (0.46-1.787) | 0.778 | 0.748 (0.378-1.478) | 0.404 |
| Temporal profiles of CRS |  |  |  |  |  |  |  |
| Onset time, days | / | 1.03 (0.913-1.162) | 0.633 | 1.005 (0.883-1.144) | 0.942 | 0.96 (0.849-1.086) | 0.52 |
| Extreme time, days | / | 0.912 (0.827-1.005) | 0.063 | 0.943 (0.863-1.031) | 0.199 | 0.971 (0.885-1.065) | 0.529 |
| Recovery time, days | / | 0.961 (0.898-1.028) | 0.245 | **0.939 (0.889-0.991)** | **0.023** | **0.948 (0.899-0.999)** | **0.047** |
| Duration from onset to recovery, days | / | 0.954 (0.889-1.024) | 0.189 | **0.94 (0.892-0.992)** | **0.023** | 0.956 (0.909-1.006) | 0.081 |
| Cytokines and serum biochemical markers |  |  |  |  |  |  |  |
| Max lg CRP, mg/L | / | **0.606 (0.39-0.94)** | **0.025** | 0.688 (0.458-1.033) | 0.072 | 0.802 (0.525-1.226) | 0.309 |
| Max lg IL-2, pg/mL | / | 0.857 (0.595-1.233) | 0.404 | 1.136 (0.824-1.566) | 0.435 | 1.064 (0.735-1.539) | 0.742 |
| Max lg IL-4, pg/mL | / | 0.77 (0.509-1.165) | 0.216 | 0.72 (0.457-1.135) | 0.157 | 1.01 (0.673-1.514) | 0.962 |
| Max lg IL-6, pg/mL | / | 0.787 (0.608-1.018) | 0.068 | 0.891 (0.701-1.132) | 0.345 | 0.857 (0.672-1.092) | 0.211 |
| Max lg IL-10, pg/mL | / | **0.614 (0.419-0.901)** | **0.013** | **0.62 (0.422-0.911)** | **0.015** | 0.736 (0.505-1.073) | 0.111 |
| Max lg IFNγ, pg/mL | / | 0.8 (0.635-1.008) | 0.058 | 0.845 (0.672-1.062) | 0.148 | 0.835 (0.665-1.047) | 0.119 |
| Max lg TNFα, pg/mL | / | 0.96 (0.688-1.338) | 0.809 | 0.925 (0.675-1.27) | 0.631 | 0.863 (0.603-1.236) | 0.421 |
| Max lg IL-17A, pg/mL | / | 0.976 (0.726-1.313) | 0.873 | 0.968 (0.741-1.264) | 0.811 | 0.906 (0.685-1.199) | 0.491 |
| Max lg ferritin, ng/mL | / | **0.315 (0.194-0.512)** | **<0.001** | **0.454 (0.298-0.692)** | **<0.001** | **0.547 (0.366-0.819)** | **0.003** |
| Max lg D-dimer, μg/L | / | **0.734 (0.552-0.976)** | **0.034** | 0.814 (0.612-1.082) | 0.156 | **0.735 (0.547-0.987)** | **0.04** |
| Baseline lg ferritin, ng/mL | / | 0.635 (0.368-1.096) | 0.103 | 0.594 (0.342-1.031) | 0.064 | 0.653 (0.389-1.095) | 0.106 |
| Baseline lg LDH, U/L | / | 0.641 (0.282-1.457) | 0.288 | 0.515 (0.222-1.192) | 0.121 | 0.635 (0.278-1.451) | 0.281 |
| Usage of tocilizumab or corticosteroids, n (%) | Yes vs. No | **0.434 (0.241-0.783)** | **0.005** | 0.71 (0.427-1.181) | 0.187 | 0.703 (0.415-1.192) | 0.191 |

Abbreviations: BMI, body mass index; CRP, C-reactive protein; CRS, cytokine release syndrome; HSCT, hematopoietic stem cell transplantation; IFN, interferon; IL, interleukin; LDH, lactate dehydrogenase; ST, severe thrombocytopenia; TNF, tumor necrosis factor.

**Supplementary Table 7. Univariate analysis of prolonged hematological toxicities after CAR-T cell infusion**

| Characteristic | Groups | Odds Ratio | P value |
| --- | --- | --- | --- |
| Age, years | / | 0.986 (0.954-1.02) | 0.416 |
| BMI, kg/m^2^ | / | 0.94 (0.806-1.096) | 0.428 |
| No. of prior chemotherapies, times | / | 1.059 (0.953-1.176) | 0.288 |
| Prior allogeneic HSCT | Yes vs. No | 1.292 (0.416-4.012) | 0.657 |
| No. of relapses, times | / | 1.208 (0.816-1.789) | 0.345 |
| Bone marrow tumor burden, % | / | **1.023 (1.005-1.04)** | **0.012** |
| Does of CAR-T cell, 10^6^/kg | / | 0.937 (0.767-1.144) | 0.523 |
| Pre-lymphodepletion |  |  |  |
| ANC, ×10^9^/L | / | 1.113 (0.865-1.432) | 0.403 |
| Hemoglobin, g/L | / | **0.977 (0.956-0.999)** | **0.042** |
| Platelet count, ×10^9^/L | / | 0.993 (0.986-1.001 ­) | 0.085 |
| Targets of CAR-T cell |  |  |  |
|  | CD19 | 1 | 0.414 |
|  | CD22 | 2.846 (0.363-22.316) | 0.319 |
|  | CD19-CD22 | 0.632 (0.18-2.217) | 0.474 |
| Neurotoxicity | Yes vs. No | 1.594 (0.357-7.11) | 0.541 |
| CRS grade |  |  |  |
|  | 0 | 1 | 0.199 |
|  | 1-2 | 4.16 (0.468-36.956) | 0.201 |
|  | 3-4 | 6.842 (0.779-60.124) | 0.083 |
| Temporal profiles of CRS |  |  |  |
| Onset time, days | / | 1.227 (0.983-1.532) | 0.07 |
| Extreme time, days | / | **1.205 (1.003-1.447)** | **0.046** |
| Recovery time, days | / | **1.149 (1.025-1.289)** | **0.017** |
| Duration from onset to recovery, days | / | 1.096 (0.997-1.205) | 0.059 |
| Cytokines and serum biochemical markers |  |  |  |
| Max lg CRP, mg/L | / | 1.598 (0.555-4.607) | 0.385 |
| Max lg IL-2, pg/mL | / | 0.503 (0.235-1.076) | 0.076 |
| Max lg IL-4, pg/mL | / | 1.57 (0.599-4.114) | 0.359 |
| Max lg IL-6, pg/mL | / | 1.061 (0.636-1.77) | 0.821 |
| Max lg IL-10, pg/mL | / | 1.661 (0.768-3.59) | 0.197 |
| Max lg IFNγ, pg/mL | / | 1.096 (0.678-1.771) | 0.708 |
| Max lg TNFα, pg/mL | / | 1.403 (0.717­-2.747) | 0.323 |
| Max lg IL-17A, pg/mL | / | 1.5 (0.706-3.185) | 0.291 |
| Max lg ferritin, ng/mL | / | **5.723 (1.788-18.32)** | **0.003** |
| Max lg D-dimer, μg/L | / | 1.522 (0.792-2.923) | 0.207 |
| Baseline lg ferritin, ng/mL | / | 2.847 (0.837-9.676) | 0.094 |
| Baseline lg LDH, U/L | / | 2.168 (0.514-9.153) | 0.292 |
| Usage of tocilizumab or corticosteroids | Yes vs. No | 1.345 (0.466-3.887) | 0.584 |

Abbreviations: BMI, body mass index; CRP, C-reactive protein; CRS, cytokine release syndrome; HSCT, hematopoietic stem cell transplantation; IFN, interferon; IL, interleukin; LDH, lactate dehydrogenase; ST, severe thrombocytopenia; TNF, tumor necrosis factor.

**Supplementary Table 8. Prolonged hematological toxicities with other adverse events after CAR-T cell infusion (n=76)**

| Characteristic | PHT (n=19) | Non-PHT (n=57) | P value |
| --- | --- | --- | --- |
| Infection, n (%) | 5 (26%) | 9 (16%) | 0.305 |
| Hemorrhage, n (%) | 7 (37%) | 6 (11%) | **0.008** |
| Non-relapse mortality, n (%) | 1 (5%) | 4 (7%) | 0.789 |

Data were described as n (%). P value were tested by Chi-Square test.

Abbreviation: PHT, prolonged hematological toxicity.

**Supplementary Figures:**


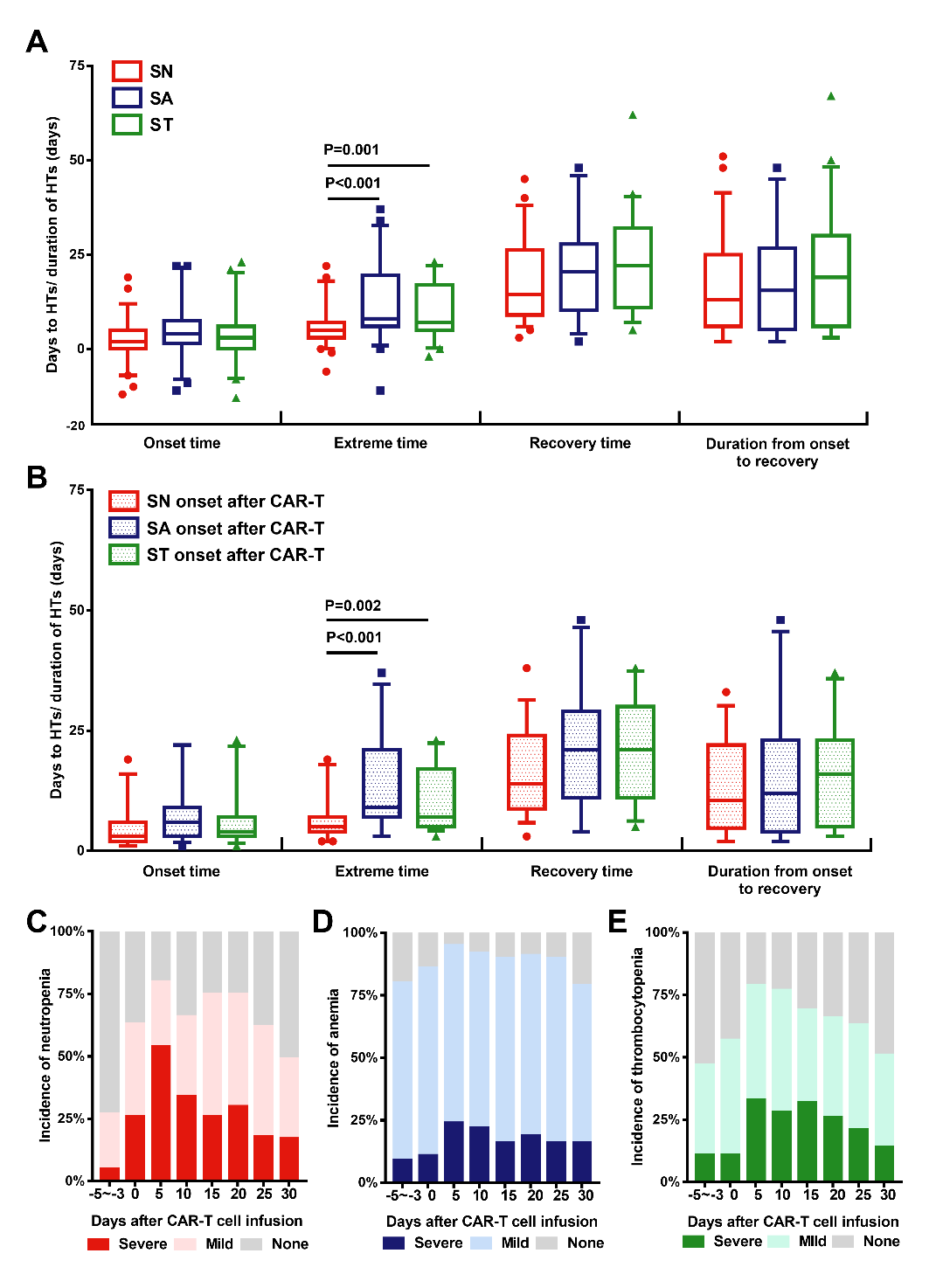


**Supplementary Figure 1.** Temporal characteristics of severe cytopenia post CAR-T cell infusion and dynamic change of incidence of cytopenia peri-CAR-T cell therapy. **(A-B)** The onset, extreme, recovery time as well as duration of severe cytopenia and new-onset severe cytopenia post CAR-T cell infusion are shown. **(C-E)** The incidences of neutropenia, anemia and thrombocytopenia are observed within a month at an interval of 5 days. Two-sided P values were determined using the Kruskal-Wallis test. HT, hematological toxicity; SN, severe neutropenia; SA, severe anemia; ST, severe thrombocytopenia.


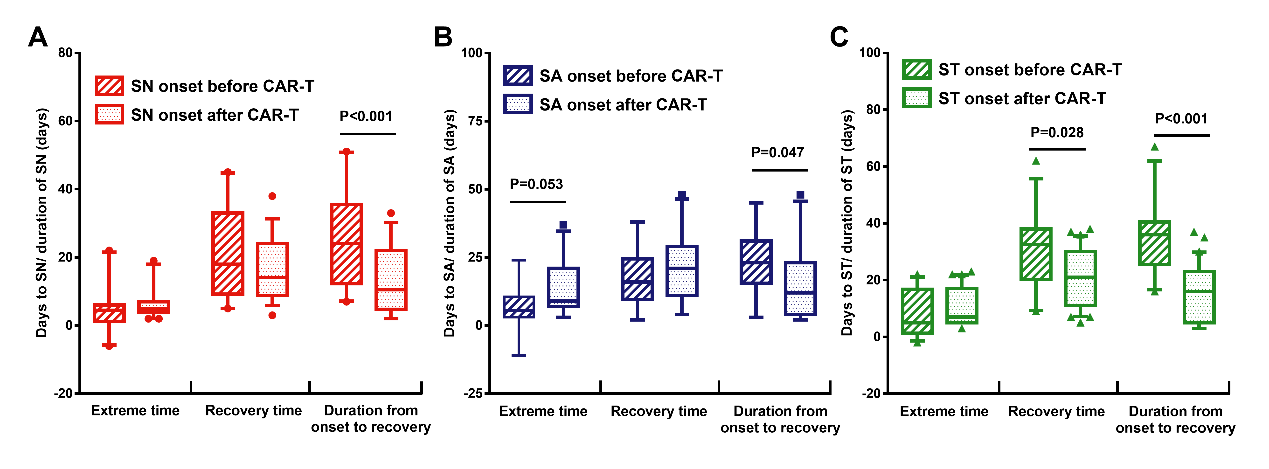


**Supplementary Figure 2.** Comparison of temporal characteristics between severe cytopenia onset before CAR-T cell infusion and new-onset severe cytopenia. The characteristics of SN, SA, and ST were concluded in **A**, **B** and **C**, respectively. Two-sided P values were determined using the Kruskal-Wallis test. SN, severe neutropenia; SA, severe anemia; ST, severe thrombocytopenia.


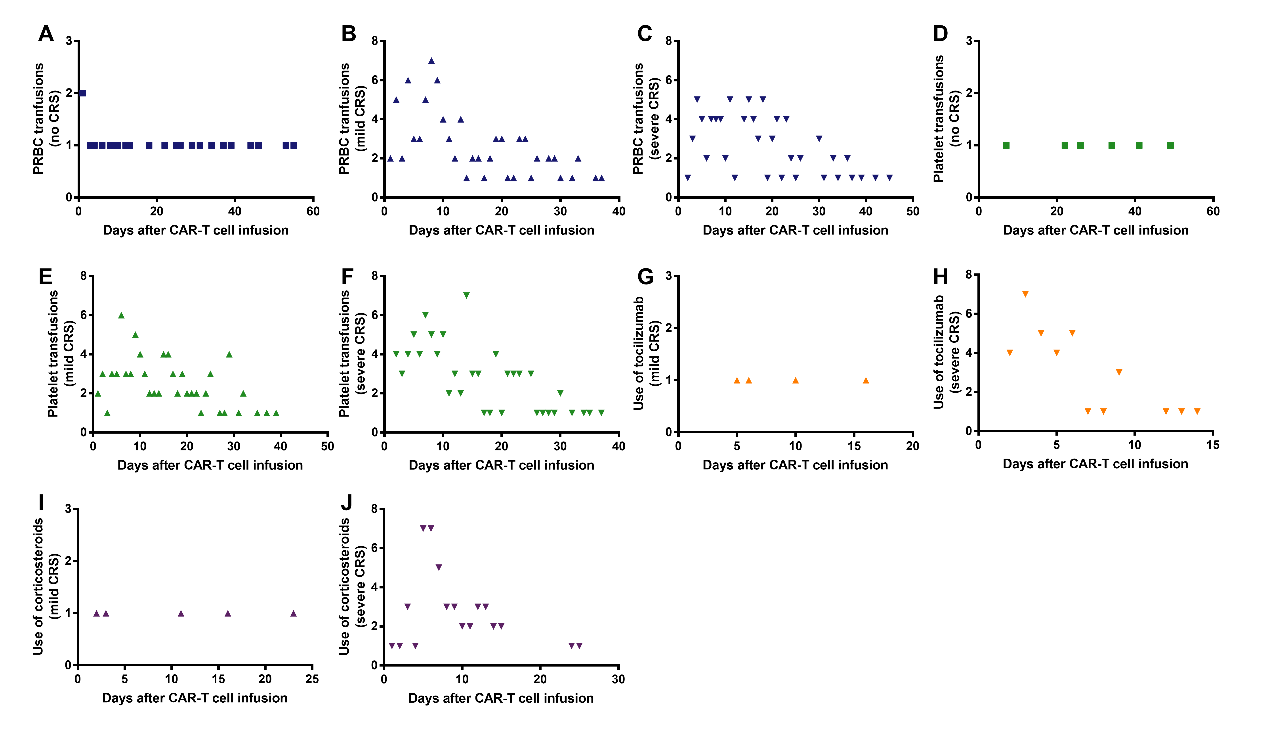


**Supplementary Figure 3.** Frequencies of blood transfusions and tocilizumab/corticosteroids use among different CRS grade groups. **(A-C)** Frequencies of PRBC transfusions in different CRS groups. **(D-F)** Frequencies of platelet transfusions in different CRS groups. **(G-J)** Frequencies of tocilizumab/corticosteroids administration in mild and severe CRS groups. CRS, cytokine release syndrome; PRBC, packed red blood cells.
